# Precision tuning of STING signaling: a mutational blueprint

**DOI:** 10.1038/s41392-026-02968-y

**Published:** 2026-09-09

**Authors:** Angelica Cuapio, Hans-Gustaf Ljunggren

**Affiliations:** Center for Infectious Medicine, Department of Medicine Huddinge, Karolinska Institutet, Stockholm, Sweden

**Keywords:** Innate immunity, Inflammation

In a recent publication in *Nature*, Zhang et al. present a comprehensive functional atlas of stimulator of interferon genes (STING), a central adaptor of innate immunity.^1^ By systematically interrogating thousands of single amino-acid substitutions, the study reveals how distinct structural regions of STING collectively regulate interferon induction, NF-κB activation and non-canonical autophagy. The work provides a new molecular framework for understanding STING activation, disease-associated variants and future therapeutic modulation of this pathway.

**Fig. 1:**
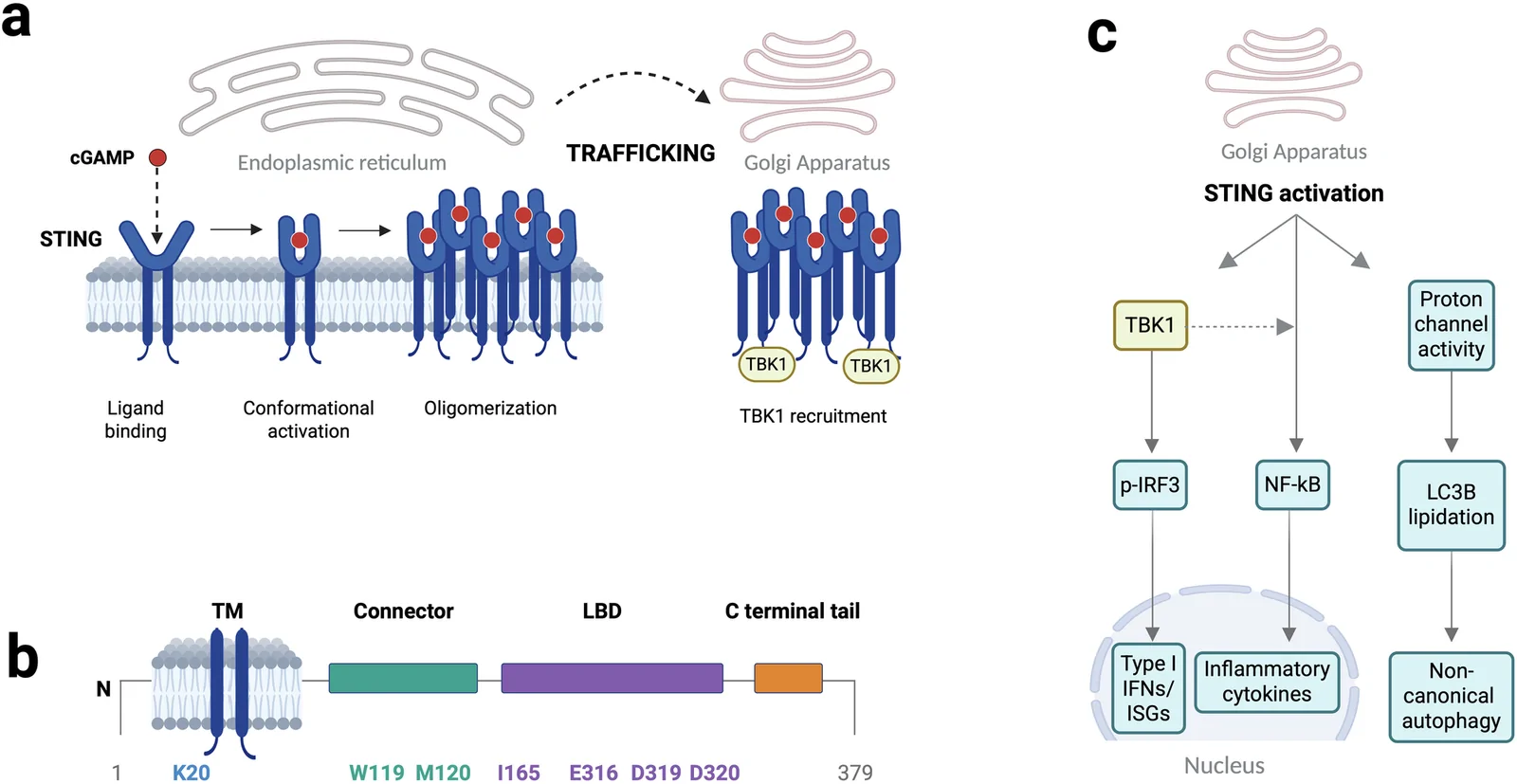
Overview of STING activation and structural regions regulating downstream signaling. **a** Binding of the endogenous cyclic dinucleotide 2′3′-cGAMP induces conformational activation of endoplasmic reticulum (ER)-resident STING dimers, promoting higher-order oligomerization. Activated STING subsequently traffics from the ER to the Golgi apparatus, where it recruits TANK-binding kinase 1 (TBK1) to initiate downstream signaling. **b** Human STING comprises an N-terminal transmembrane (TM) region, a connector region, a cytosolic ligand-binding domain (LBD), and a C-terminal tail. The highlighted amino acid residues (K20, W119, M120, I165, E316, D319 and D320) represent key regulatory sites identified by comprehensive deep mutational scanning. Single amino acid-substitutions at these positions can markedly alter STING activity by promoting constitutive activation, enhancing responsiveness to ligand, or altering downstream signaling outputs. **c** Following trafficking to the Golgi apparatus, activated STING initiates three principal signaling branches. Recruitment of TBK1 leads to phosphorylation and activation of IRF3, inducing expression of type I interferons (IFNs) and interferon-stimulated genes (ISGs). In parallel, activated STING stimulates NF-κB-dependent inflammatory gene expression. Independently, STING functions as a proton channel in Golgi/post-Golgi membranes, promoting LC3B lipidation and induction of non-canonical autophagy. Illustration created with BioRender.com

STING occupies a central position in innate immune sensing. It functions as the central adaptor downstream of cyclic GMP–AMP synthase (cGAS), which detects cytosolic DNA and generates the second messenger 2′3′-cGAMP. Binding of 2′3′-cGAMP induces large conformational rearrangements, oligomerization and trafficking from the endoplasmic reticulum to Golgi and post-Golgi compartments. These events trigger recruitment and activation of TBK1, leading to phosphorylation of IRF3, resulting in type I interferon production, and also promote NF-κB-dependent inflammatory responses. In parallel, STING can induce a non-canonical form of autophagy, linked to LC3B lipidation and, as recently shown, proton-channel activity in post-Golgi membranes.^2–4^ Through these outputs, STING contributes to host defense against infection, immune surveillance of cancer, cellular senescence and inflammatory disease. Its central position has made STING an attractive therapeutic target, with efforts directed both toward agonists, for cancer immunotherapy and vaccine adjuvanticity, and antagonists, for interferonopathies and other inflammatory conditions. Yet STING signaling is not a simple on–off switch. It involves ligand binding, conformational transition, higher-order assembly, intracellular trafficking and pathway-specific effector recruitment. How these processes are encoded within the primary sequence of STING has remained incompletely understood (Fig. 1).

Zhang et al. addressed this question by combining deep mutational scanning with cellular phenotyping, structural biology and clinical variant interpretation. The authors generated a saturation mutagenesis library covering nearly every possible single amino-acid substitution in human STING and introduced these variants into reporter systems designed to quantify pathway activation. Initially, they screened for variants able to induce type I interferon signaling in the absence of ligand, thereby identifying constitutively active forms of STING. They then extended the analysis to cGAMP-induced activation and to LC3B lipidation, allowing them to distinguish mutations that affected transcriptional and non-transcriptional STING outputs. Selected variants were examined in greater mechanistic depth using immunoblotting, microscopy, RNA sequencing in STING-deficient THP-1 cells, and cryo-electron microscopy. This experimental design allowed the authors not only to identify gain- and loss-of-function mutations, but also to classify mutations according to the signaling they produced. Importantly, the approach moved beyond previously known SAVI-associated mutations and canonical ligand-binding residues, uncovering unexpected regulatory elements distributed across the transmembrane region, luminal loops, ligand-binding domain and C-terminal regions of STING.

Several important conclusions emerge from the study. First, STING is restrained by a distributed network of autoinhibitory structural elements. Mutations at sites such as K20, W119/M120, I165 and E316/D319/D320 can release these constraints and promote ligand-independent signaling. Cryo-EM analyses showed that different hyperactive variants adopt distinct oligomeric geometries, indicating that alternative structural routes can converge on signaling competence. Second, STING outputs can be uncoupled. Some mutations preserved interferon induction while impairing LC3B lipidation, whereas others altered the balance between interferon and NF-κB activity. This demonstrates that STING signaling comprises distinct downstream programs that can be independently tuned, rather than merely active or inactive. Thus, the work shifts the view of STING from a binary signaling switch to a multidimensional signaling platform whose output can be selectively tuned by discrete structural perturbations. Third, the study provides a powerful interpretive framework for human genetic variation. Functional scores helped distinguish benign from pathogenic variants, identified a rare D319G variant associated with SAVI-like disease, and revealed that cancer-associated STING mutations are enriched for loss-of-function effects, consistent with selection against DNA-triggered immune surveillance. Together, these findings redefine STING as a finely balanced signaling machinery in which single amino-acid substitutions can alter ligand sensitivity, intracellular trafficking, oligomerization and pathway choice.

The implications are broad. For basic immunology, the study provides a sequence-level grammar of STING activation and shows how conformational mechanics are translated into distinct immune outputs. For human genetics, it offers a functional roadmap for interpreting variants of uncertain significance in autoinflammatory disease and cancer. For drug development, it suggests that future STING agonists and antagonists may be designed not only to enhance or inhibit signaling, but to bias signaling toward selected downstream programs. Such pathway-selective modulation could be particularly valuable in cancer, infection, vaccine development and inflammatory disease. More broadly, the work exemplifies how comprehensive sequence-to-function maps can bridge structural biology, human genetics, and therapeutic discovery for immune signaling proteins. As similar approaches are extended across innate immunity, they are likely to reshape how signaling pathways are understood, disease-associated variants are interpreted, and next-generation immunomodulatory therapies are developed. Beyond STING biology, this study illustrates how comprehensive sequence-to-function mapping can transform our understanding of dynamic signaling proteins, providing mechanistic insight while simultaneously informing clinical interpretation and therapeutic design.
